# Targeting CD137 (4-1BB) towards improved safety and efficacy for cancer immunotherapy

**DOI:** 10.3389/fimmu.2023.1208788

**Published:** 2023-06-02

**Authors:** Guizhong Liu, Peter Luo

**Affiliations:** ^1^ Adagene Inc., San Diego, CA, United States; ^2^ Adagene (Suzhou) Limited., Suzhou, China

**Keywords:** TNFR agonist, CD137/4-1BB, FcγR mediated cross-linking, conditional activation, cancer immunotherapy, costimulatory receptor

## Abstract

T cells play a critical role in antitumor immunity, where T cell activation is regulated by both inhibitory and costimulatory receptor signaling that fine-tune T cell activity during different stages of T cell immune responses. Currently, cancer immunotherapy by targeting inhibitory receptors such as CTLA-4 and PD-1/L1, and their combination by antagonist antibodies, has been well established. However, developing agonist antibodies that target costimulatory receptors such as CD28 and CD137/4-1BB has faced considerable challenges, including highly publicized adverse events. Intracellular costimulatory domains of CD28 and/or CD137/4-1BB are essential for the clinical benefits of FDA-approved chimeric antigen receptor T cell (CAR-T) therapies. The major challenge is how to decouple efficacy from toxicity by systemic immune activation. This review focuses on anti-CD137 agonist monoclonal antibodies with different IgG isotypes in clinical development. It discusses CD137 biology in the context of anti-CD137 agonist drug discovery, including the binding epitope selected for anti-CD137 agonist antibody in competition or not with CD137 ligand (CD137L), the IgG isotype of antibodies selected with an impact on crosslinking by Fc gamma receptors, and the conditional activation of anti-CD137 antibodies for safe and potent engagement with CD137 in the tumor microenvironment (TME). We discuss and compare the potential mechanisms/effects of different CD137 targeting strategies and agents under development and how rational combinations could enhance antitumor activities without amplifying the toxicity of these agonist antibodies.

## Introduction

T cell-mediated immunity is crucial for the host antitumor response (1). Under physiological conditions, T cell activation requires two signals: signal 1 involves TCR activation triggered by the major histocompatibility complex (MHC) presented antigenic peptide, and signal 2, a costimulatory signal, amplifies the antigen-specific signal 1 (2). CD137 or 4-1BB, a member of the tumor necrosis factor receptor superfamily (TNFRSF), also known as TNFRSF9, is one of the key costimulatory receptors identified that has shown promise as a therapeutic target for boosting antitumor immune responses in both preclinical and clinical studies over the past two decades (3). CD137 is induced upon activation in T cells, B cells, and natural killer (NK) cells (4). Ligation of CD137 by its natural ligand, CD137L or 4-1BBL, recruits TNFR-associated factor (TRAF) 1 and TRAF2 and induces signaling through the master transcription factor NF-kB and MAPKs (5) (6), which coactivates CD8+ T cells and natural killer cells, resulting in enhanced cellular proliferation and survival, increased proinflammatory cytokine secretion, cytolytic function, and antibody-dependent cell-mediated cytotoxicity (7). As most tumors are killed by CTLs in an antigen-specific manner, agents that propel CD8+ T-cell activation and impart strong cytolytic, inflammatory, and immune-regulating properties in an antigen-specific manner are ideal candidates for enhancing antitumor immunity. Agonistic anti-CD137 mAb immunotherapy targeting CD8+ T cells meets these requisites. However, clinical development of the first fully human anti-CD137 IgG4 agonistic antibody, urelumab, was put on hold due to dose-dependent liver toxicity, including grade 3 and higher liver-related toxicities and two fatalities, despite demonstrating monotherapy efficacy in melanoma patients (3) (8). By contrast, the second clinical development of a fully human IgG2 anti-CD137 agonist antibody, utomilumab, has shown excellent safety and tolerability following a lengthy dose escalation schedule, presumably, due to the clinical safety issues for urelumab, the first agonist anti-CD137 antibody in clinics. Only modest or marginal efficacy in utomilumab monotherapy is reported for a few patients in immune-responsive Merkel cell carcinoma and for checkpoint-experienced melanoma and non-small cell lung cancer patients (9). These two extreme cases highlight the challenges in developing costimulatory receptor antibody therapies in general and anti-CD137 agonist antibodies in particular, where the preclinical observation is yet to translate into clinical reality. New generations of CD137 agonists with different targeting strategies are under development that could get around these challenges and realize the full potential of CD137 targeted immunotherapy for cancer treatment.

## CD137 binding epitopes, complex conformations and IgG isotypes

**Figure 1:**
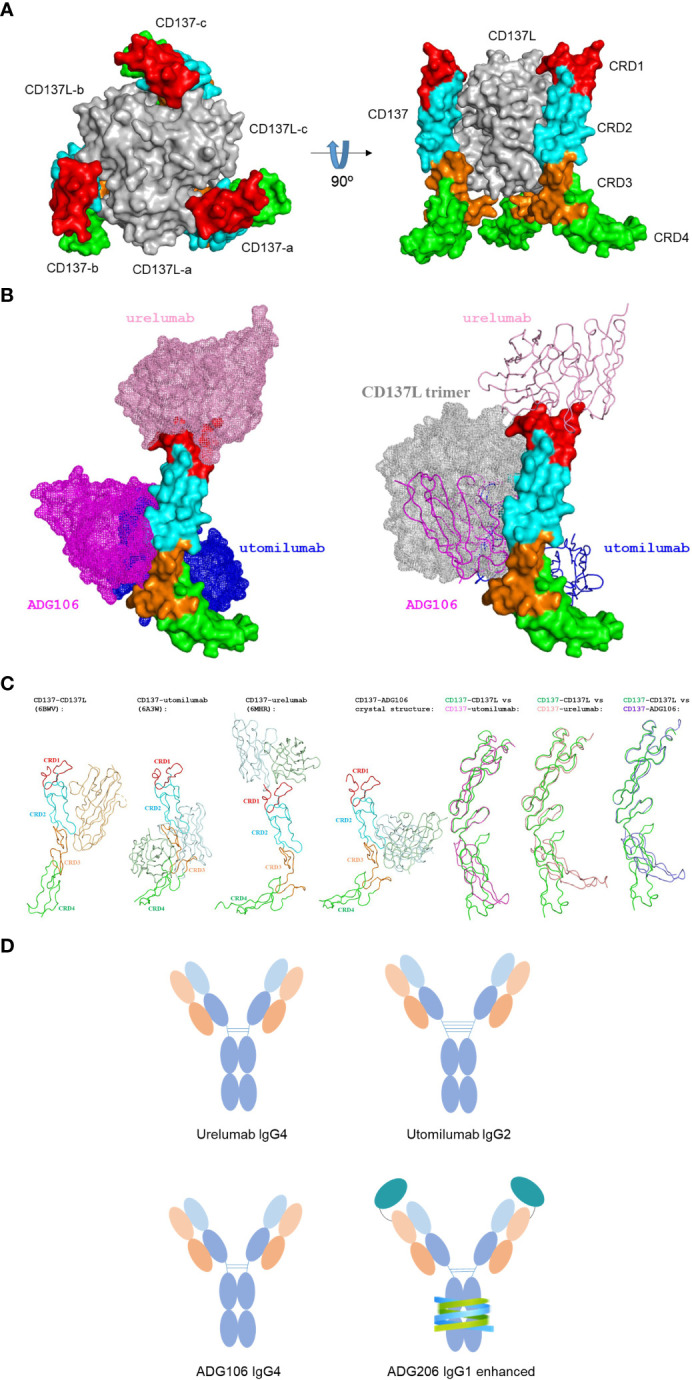
**(A)** The constructed structure of trimeric CD137 in complex with trimeric CD137L; **(B)** Structure of CD137 in complex with anti-CD137 agonistic mAbs with only one CD137 structure shown to illustrate the difference in CD137 binding epitopes by 3 anti-CD137 agonistic mAbs, together to show the complete overlap between ADG106 with CD137L trimers in gray; **(C)** The induced conformational changes of CD137 upon binding to CD137 ligand, three agonist antibodies and their pairwise comparison of the induced conformational change of CD137 with reference to its conformation upon binding to CD137 ligand; **(D)** the diagram illustrating the difference of IgG isotypes for urelumab, utomilumab, ADG106 and ADG206.

The binding epitope on CD137 of an anti-CD137 antibody could have direct impact on its agonistic activity. **Figure 1A** illustrates that the trimeric CD137 ligand in gray binds to CRD2 and 3 on CD137 to cluster the receptor, whereas the binding epitopes of different anti-CD137 agonist antibodies can vary (10). For example, Urelumab binds to the N-terminal portion of CRD-1, utomilumab binds at the junction of CRD-3 and CRD-4 (10), and ADG106, a fully human anti-CD137 IgG4 agonist antibody developed by us, binds at the junction of CRD-2 and CRD-3, which overlaps with the CD137L binding site at CRD-2 and CRD-3 (**Figure 1B**) (11). Such differences in binding epitopes among different agonistic anti-CD137 antibodies explain their ligand-blocking versus non-blocking properties. Urelumab does not block CD137L interaction with CD137, whereas ADG106 strongly blocks CD137 binding to its ligand.

It should be noted that the conformation of the CD137 complex also depends on the binding epitope of the agonist antibodies in comparison with the CD137 ligand-induced complex conformation, which may play a role in CD137-mediated T cell signaling. CD137 conformations in complex with different agonist antibodies, including urelumab, utomilumab, and ADG106, in comparison with CD137 ligand seem to follow a trend that mimics their functional activities.

The CD137 binding epitope may also affect the antibody’s Fc interactions with FcγRs. Such variations in epitope-oriented FcγRs engagement could further contribute to differences in the levels of agonist activity for different anti-CD137 agonistic mAbs. In addition, the difference in IgG isotypes, because of their importance in FcγRs, especially FcγRIIB-mediated crosslinking for agonistic activities, is also shown in **Figure 1D**, for urelumab in IgG4, utomilumab in IgG2, ADG106 in IgG4, and ADG206, a conditionally activatable ADG106 in Fc-enhanced IgG1 (more details in the following sections).

Moreover, the interactions between CD137 and CD137L not only trigger CD137-mediated costimulatory signaling, but a reverse signaling through CD137L may also be activated to regulate immune responses (12). Previous studies have indicated that blockade of CD137L reverse signaling promotes intra-tumoral differentiation of IFNγ-producing cytotoxic T cells, IL12-producing CD103+ DC, and type 1 tumor-associated macrophages to suppress tumor growth (13). Thus, the ligand-blocking anti-CD137 antibody could have additional pharmacological activities through inhibiting the CD137L-mediated reverse signaling. The ligand-blocking versus non-blocking properties of the agonist antibodies can also impact their activity in the presence and absence of CD137 ligand. For example, as a ligand non-blocking agonist, urelumab was demonstrated to be capable of inducing strong ligand-dependent CD137 clustering through cross-linking receptors trimerized by the ligand, whereas the ligand-blocking utomilumab failed to induce ligand-dependent clustering (10). Thus, the influence on ligand/receptor interaction could be a noteworthy property of an anti-CD137 agonist antibody when comprehensively evaluating its pharmacological activity.

Finally, we noted that CRD4 is tilted by Utomilumab-induced conformational change, while CRD4 is much more tilted by urelumab and ADG106, respectively, in comparison with the reference state by CD137L. This becomes quite obvious once we overlap their CD137 structures in complex with different agonist antibodies with that of CD137 (in green) in complex with CD137L based on the CRD1 and CRD2 as shown in **Figure 1C**.

## Crosslinking by FcγR

**Figure 2:**
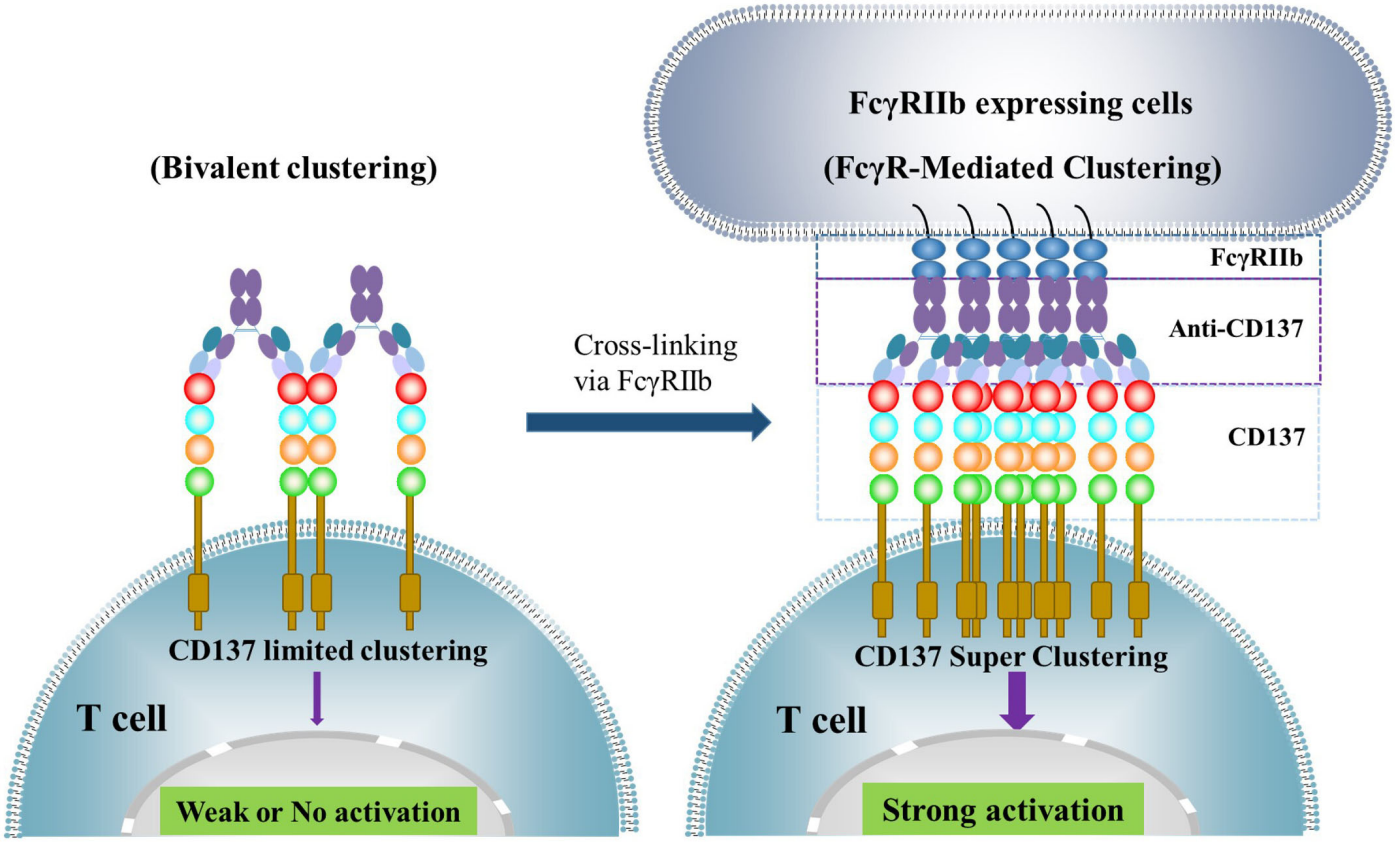
illustrates how the activation of CD137 receptor signaling is achieved through anti-CD137 agonistic monoclonal antibodies (mAbs). These antibodies bind to CD137 through their Fab region, inducing limited clustering of CD137 receptors and weak or no activation of downstream signaling. However, when the Fc region binds to FcγRs, particularly FcγRIIb, expressed on cells such as dendritic cells, macrophages, and B cells, they can induce CD137 receptor super-clustering and strong downstream signaling activation.

In physiological conditions, CD137 costimulatory signaling activation requires clustering of the receptor through its natural ligand to form trimeric or larger lattice-shaped structures (14). Similarly, agonistic antibodies activate CD137 receptor signaling by inducing cluster formation at high receptor density (15). However, the crystal structure of utomilumab in complex with CD137 indicates that utomilumab binds to monomeric or dimeric CD137 to induce limited crosslinking of the receptor (16). This explains why the Fab binding to CD137 alone by many anti-CD137 monoclonal antibodies (mAbs) is not sufficient to induce CD137 receptor activation. Most anti-CD137 agonistic antibodies require engagement of FcγRs, particularly FcγRIIb, to mediate clustering and activation of CD137. Upon Fc binding to FcγRIIb, higher order CD137 receptor clusters can be achieved, which activates CD137-mediated downstream signaling (**Figure 2**). Certain antibodies, such as urelumab, can directly activate CD137 receptor independent of FcγR engagement. A study comparing the agonist activity of urelumab, utomilumab, and AD106 in the CD137 Jurkat NFκB reporter cell signaling assay in the presence and absence of FcγRIIb-expressing cells demonstrated that, in the presence of FcγRIIb-expressing cells, all three antibodies stimulate CD137 and downstream signaling, with urelumab showing more potent activity than utomilumab and ADG106. However, in the absence of FcγRIIb-expressing cells, both utomilumab and ADG106 are inactive, whereas urelumab is still capable of stimulating CD137 receptor activation (11). These results suggest that the binding epitope of urelumab may allow this antibody to cluster CD137 monomers or dimers more efficiently through bivalent binding than other anti-CD137 agonists. Nonetheless, engagement of FcγRs, particularly FcγRIIb, by Fc could further enhance the clustering effect (17) (18). FcγRIIb is expressed on many types of immune cells, including B cells, dendritic cells (DCs), monocytes/macrophages, mast cells, and basophils (19). Notably, FcγRIIb is expressed on Kupffer cells, the resident liver macrophages, and liver sinusoidal endothelial cells in the liver, where it plays an important role in immune complex clearance (20). This may significantly contribute to the super-clustering and hyperactivation of CD137 by anti-CD137 agonists on immune cells in the liver, leading to hepatic inflammation and liver toxicity, as exemplified by urelumab (8).

It has been shown that the non-blocking ligand urelumab increases the clustering of CD137 receptors on cells in a ligand-dependent manner (10). This may explain how urelumab stimulates CD137 receptor activation in the absence of FcγR engagement and cause severe toxicity, particularly liver damage, in patients. Conversely, the mild agonistic activity of utomilumab may account for its weak clinical efficacy in patients.

To strengthen the activity of CD137 agonist antibodies, enhancing the clustering of CD137 receptors has been a strategy, especially for those anti-CD137 mAbs with weaker agonist activity. Fc engineering can achieve this by enhancing crosslinking by FcγRIIb. Examples include LVGN6051 (21) in an IgG4 backbone and ADG206 (22) in an IgG1 backbone. ADG206 has shown four-fold higher cross-linking potency than urelumab in the functional assay, where urelumab is the most potent anti-CD137 agonist shown clinically.

Notably, CD137 is not only expressed in activated effector T cells but has also been identified as an activation marker for antigen-specific T regulatory (Treg) cells with immunosuppressive functions (23). CD137+ cells form a major part of functional tumor Tregs (24). In one preclinical study using mouse tumor models, two mechanisms were shown to promote robust tumor rejection: tumor Treg depletion and effector T cell agonism by anti-CD137 mAbs, which, however, are competitive and dependent on antibody isotype and FcγR availability. Administration of anti-CD137 mouse IgG2a, which preferentially depletes Treg cells, followed by either agonistic anti-CD137 mouse IgG1 or anti-PD-1 mAb augmented anti-tumor responses. An antibody engineered to optimize both FcγR-dependent Treg cell depleting capacity and FcγR-independent agonism delivered enhanced anti-tumor therapy (25). Although none of the current clinical stage anti-CD137 agonist antibodies developed so far demonstrated Treg depleting capacity, such mechanism can be provided by a Treg depleting antibody, such as anti-CTLA-4 mAb (26) (27), in a combination setting.

## Binding affinity and agonism

High affinity is often the desired characteristic for therapeutic antibodies, particularly for antagonistic antibodies that can neutralize or inhibit target functions. However, for agonistic antibodies, this is not always the case. Epitope and Fc-mediated crosslinking, rather than high affinity, are critical for the antitumor activity of CD137 agonist antibodies with reduced liver toxicity (28). A recent study examined the relationship between affinity and function of immunomodulatory antibodies and found that reducing affinity can be a strategy to enhance immunomodulatory antibody agonism (29). Low rather than high affinity delivers greater activity through increased receptor clustering, independent of FcγRs. The study suggests that a faster dissociation rate, or higher off-rate rather than on-rate of the monoclonal antibody, is responsible for the increased agonistic activity of low-affinity variants. Additionally, an inert anti-CD137 mAb, utomilumab, can be transformed into an agonist. Low-affinity variants of the clinical antagonistic anti-PD-1 mAb, nivolumab, also mediated more potent signaling and affected T cell activation. Notably, low-affinity targeting by antibody binding is conducive to receptor activation but detrimental to Fc-mediated effector function, which could limit further enhanced receptor clustering through antibody engagement of FcγRs. Nonetheless, these findings provide a new avenue for agonistic antibody engineering (29).

## Conditional activation

**Figure 3:**
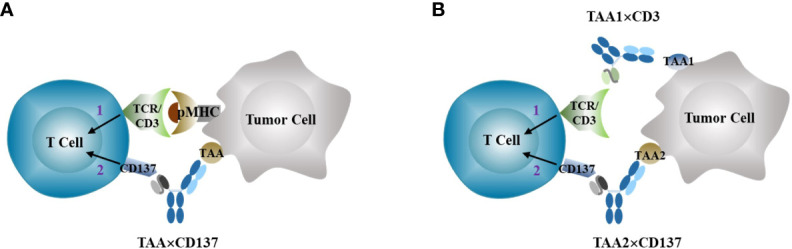
Anti-CD137 agonists (TAA×CD137 as example here) stimulates costimulatory signal 2 to enhance tumor-specific T cell activation primed by signal 1 through either neoantigen stimulated TCR activation **(A)**, or bispecific TAA×CD3 T cell engagers **(B)**.

The specificity of T cell immune responses to an antigen comes from the recognition of the antigen peptide MHC complex by TCR, and not from costimulatory signaling. However, the use of CD137 agonists for cancer immunotherapy can potentially boost T cell responses triggered by any existing antigen MHC complex, including those involved in mediating autoimmune responses. This creates a dilemma between antitumor efficacy and autoimmune toxicity for costimulatory agonists with systemic agonistic activity, such as anti-CD137 agonist antibody. To address this issue, various strategies have been developed to create next-generation anti-CD137 agonists that target the agonistic activity more specifically to the tumor site while limiting the agonistic activity in normal tissues to reduce immune-toxicity (30). The primary solution is to exploit the differences between tumors and normal tissues to target tumors more specifically. Tumor-associated antigens (TAAs) are overexpressed in tumors compared to most normal tissues, and this can be leveraged to direct the antibody primarily to tumor tissues (**Figure 3**). Several TAAs, such as HER2 (31), FAP (32), EGFR (33) (34), Claudin18.2 (35), PSMA (36), Nectin-4 (37), Mesothelin (38), and B7-H3 (39), have been selected to construct bi- or tri-specific CD137 agonistic antibodies. These antibodies directly target the tumor-associated antigens through TAA-targeting arms and provide a costimulatory signal to enhance antitumor immune responses. The agonist activity of these constructs is dependent on TAA for crosslinking, thereby limiting immune activation primarily in tumor tissues with high levels of TAA expression. A comprehensive summary of the status of development and available clinical results of these TAA×CD137 constructs has been provided in recent reviews (40) (41).

**Figure 4:**
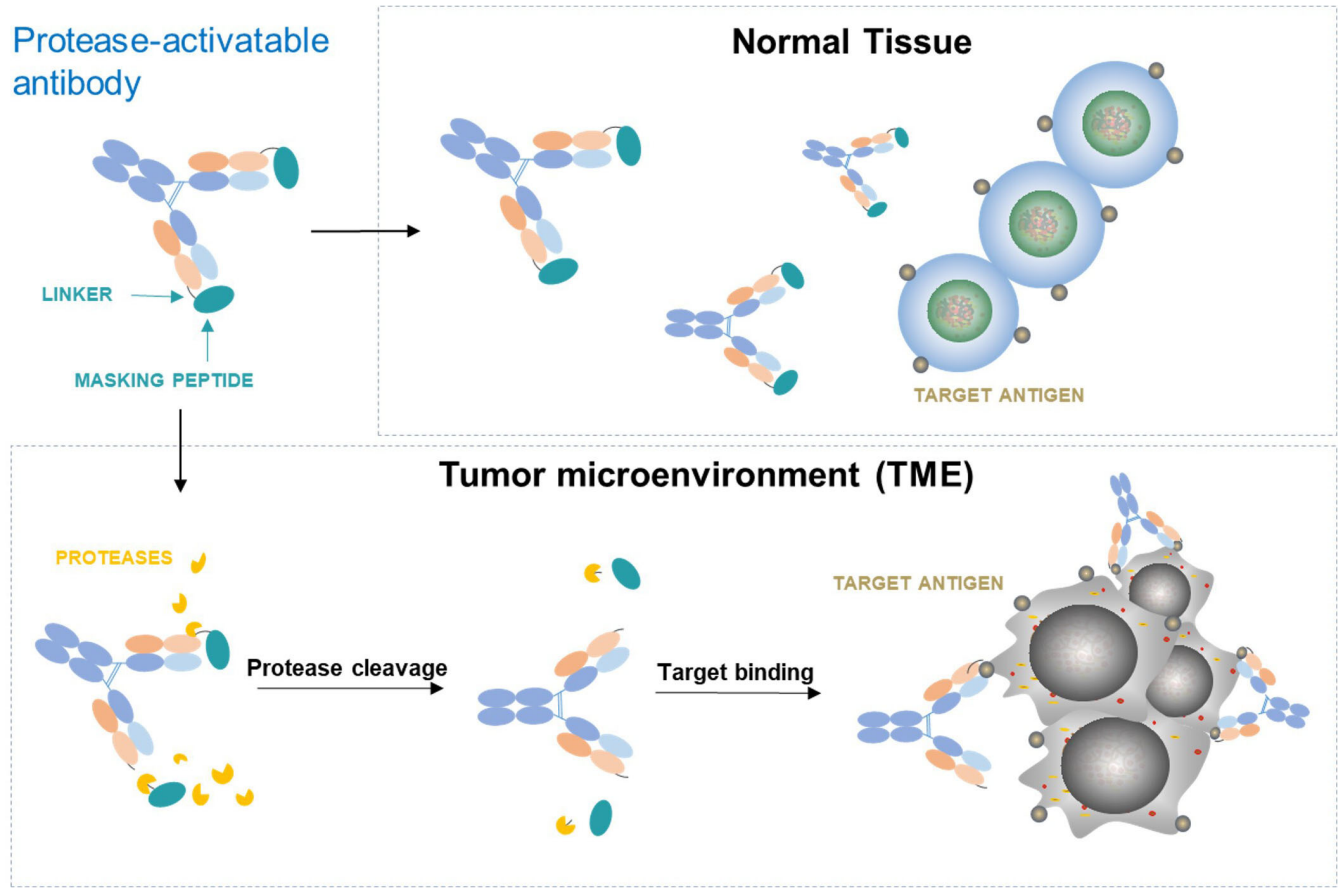
Conditionally activatable agonists controlled by antibody masking and protease-cleavable linker.

Another major difference between tumors and normal tissues is that protease activity is generally up-regulated in tumor microenvironment through upregulation of protease expression, activation of zymogen, down-modulation of inhibitor expression, or a combination of these effects (42). Multiple extracellular proteases such as matrix metalloproteinases (MMPs), urokinase-type plasminogen activator (uPA), matriptase, etc. are widely overexpressed in different tumors but with limited expression in normal tissues. These proteases play important roles in many aspects of tumor biologic processes involved in tumor signaling, angiogenesis, tumor growth and metastasis (43). To take advantage of the rich protease activity in tumors, one strategy is to design a conditionally activatable agonist by blocking the antigen binding site of the antibody with a mask that is covalently attached to the antibody through a protease cleavable linker. In such antibody configuration, the masked agonist remains largely inactive in circulation and normal tissues. However, when in tumor microenvironment (TME), the masked antibody can be enriched by binding with upregulated antigen and then permanently activated through removal of masking peptides by rich proteases in the TME to expose the binding site of the antibody. This allows activation of the agonist preferentially in tumor (**Figure 4**). Such strategy has been tested successfully in both preclinical and clinical settings for anti-CTLA-4 antagonist antibody ADG126 (42). It has been shown, for example, a masked anti-murine CD137 agonist (Pb-Tx 1D8) has been generated with antitumor efficacy and reduced toxicity in mouse models (44). Recently, ADG206, a protease-activatable masked species cross-reactive anti-CD137 with enhanced Fc crosslinking (22), has entered clinical investigation (NCT05614258), which would provide direct assessment of the masking strategy in the costimulatory agonist therapeutics. Other approaches taking advantage of the differences between tumors and normal tissues to achieve preferential tumor activation have also been explored. For example, a conditional anti-CD137 agonistic antibody STA551 that binds and crosslink CD137 in ATP-rich condition in tumor microenvironment has been generated with improved safety in human CD137 knock-in mouse models (45).

## Combinations

To date, more than 20 different CD137-targeted agonistic antibodies have been developed and entered in clinical trials (40) (41). Although these trials have demonstrated various clinical responses, the overall activity in general is modest by monotherapy with these agonists. Combinations with other therapeutics are under testing, and anti-PD-1/L1 is the most common partner with the anti-CD137 agonists in the clinical investigations and improved therapeutic outcomes have been observed (3). In addition to the traditional drug-drug combinations, one other simple strategy for combination is to construct bispecific or multi-specific therapeutics, which sometimes may achieve greater activity than that of the individual drug-drug combinations. These bi- or multi-specifics targeting both CD137 and other immune-related pathways are under development and early clinical testing, such as CD137 with OX-40 (46), CD40 (47), PD-1 (48), PD-L1 (49–51), CD47 (52). Of note, various PD-L1 × CD137 bispecific antibodies have been developed. PD-L1 was used both to target PD-1 checkpoint pathway and agonistically crosslink CD137 in TME. Clinical results from the bispecific PD-L1 × CD137 antibody GEN1046 have shown signs of T cell activation and monotherapy antitumor activity in patients refractory to anti-PD-1 therapy, with 20% patients developing controllable abnormalities in liver function test (53).

The potential of the anti-CD137 and anti-CTLA-4 therapies to synergistically treat tumors has been well-established in preclinical models and confirmed by clinical trials using cross-reactive therapeutic antibodies against both targets. Combining these therapies is not only biologically compelling for their anti-tumor efficacy but also to reduce the toxicity of each monotherapy, making the anti-CD137/anti-CTLA-4 combination the top choice for clinical translation. Anti-CD137 agonists such as ADG106/206 and anti-CTLA-4 antagonists such as ADG116/ADG126, which are related to the CD137 and CTLA-4 pathways, have shown promise in preclinical studies and are being evaluated in clinical trials (11, 22, 54, 55).

Other therapeutics that can take advantage of the T cell costimulatory activity of CD137 agonists include vaccines, certain chemotherapies, and/or radiotherapy. Costimulatory signals are required for the generation of a robust and long-term T cell response, but the target specificity of this response is determined by the TCR/pMHC recognition, also known as the priming signal or Signal 1. Combining a cancer vaccine that can initiate the tumor-specific priming signal with the costimulatory agonists is a conceptually appealing approach to enhance the targeted antitumor immune response. This approach has been evaluated in mouse tumor models with promising results, showing that CD137 agonist antibodies have unique potential to promote durable regression of HPV+ tumors when combined with an E6/E7 peptide vaccine (56). Multiple personalized or neoantigen cancer vaccines have entered clinical investigations in combination with checkpoint inhibitors, mostly anti-PD-(L)1, to overcome resistance to immune checkpoint inhibitors (57, 58). RNA-based tumor vaccines in combination with checkpoint inhibitors have also demonstrated durable objective responses in anti-PD1 experienced patients with unresectable melanoma, accompanied by the induction of strong CD4+ and CD8+ T cell immunity against the vaccine antigens (59). It is yet to be seen but worth exploring the combination of cancer vaccines and anti-CD137 agonists in a clinical setting.

CD3 bispecific T cell engagers (TCE) can directly trigger Signal 1 and combining them with the anti-CD137 agonist as Signal 2 can overcome resistance to bispecific TCE treatment in T cell-cold solid tumors in preclinical animal tumor models (60), as conceptually illustrated in **Figure 3B**.

## Translational research

Since Melero et al. first reported that agonist anti-CD137 monoclonal antibodies can eradicate transplanted mouse tumors through enhanced CD8+ T-cell antitumor immunity over two decades ago (61), extensive studies have been conducted to evaluate the antitumor activity, toxicity, and mechanisms of anti-CD137 agonists and their combinations with other agents using syngeneic mouse tumor models, which greatly facilitated our understanding of CD137-targeted immunotherapies (55). These studies either involved mouse CD137 surrogate antibodies in wild-type mouse background or human CD137 agonists but in human CD137 knock-in mouse background due to the lack of mouse cross-reactivity of many human CD137 agonist antibodies. One caveat is that the human CD137 knock-in mice may not fully recapitulate CD137 biology, as the mouse CD137 ligand does not bind to human CD137 (62). Interestingly, ADG106, as well as its conditionally activatable, Fc-enhanced form ADG206, bind to a conserved epitope of CD137 from human, monkey, and rodents, allowing direct assessment of their pharmacological/toxicological properties in wild-type mice, which may better mimic clinical conditions. In preclinical studies, ADG106 induces robust single-agent antitumor responses in multiple syngeneic tumor models and synergizes with anti-PD-(L)1 or anti-CTLA-4 checkpoint inhibitors, without showing significant toxicity (11) (22), which translated to patient studies. In clinical trials, ADG106 stimulates CD4+ and CD8+ T-cell and NK cell proliferation and proinflammatory interferon-gamma (IFN-γ) release while displaying a low risk for adverse immune responses. Notably, ADG106 treatment alone induces significant dose-dependent increases of soluble CD137, which is further enhanced when combined with anti-PD-1 antibody (63). Similar results were observed in other anti-CD137 studies and demonstrated that soluble CD137 can be a dynamic biomarker to monitor agonist CD137 immunotherapies (9) (64).

## Concluding remarks

Current checkpoint blockade therapies have shown impressive benefits in cancer treatment, but only for a minority of patients. However, recent years have seen a shift towards exploring immuno-oncology agents beyond PD1/PDL1 inhibitors in clinical trials (65). There is a pressing need for agents that can further enhance T cell immunity for cancer treatment while also having improved safety profiles. Targeting CD137, a potent T cell costimulatory receptor, with agonist antibodies shows promise for cancer immunotherapy beyond its successful application in CAR T cells (66). It is encouraging to see that multiple strategies have been employed to develop the next generation of anti-CD137 agonists that have strong agonistic activity, increased tumor specificity, and reduced agonism in normal tissues to minimize immunotoxicity. Furthermore, the success of costimulatory CD137 agonists depends on their synergistic and rational combinations with other therapeutics or CD137-based bi- or multi-specific antibodies. With many of these new agents entering clinical investigations, the next few years hold great promise for CD137-targeted immunotherapies.

## Author contributions

All authors listed have made a substantial, direct and intellectual contribution to the work, and approved it for publication.
